# Techno−Economic Analysis and Multi‐Objective Optimization of Cross‐Flow Wind Turbines for Smart Building Energy Systems

**DOI:** 10.1002/gch2.202200203

**Published:** 2023-03-03

**Authors:** Zahra Sefidgar, Ali Ashrafizadeh, Ahmad Arabkoohsar

**Affiliations:** ^1^ Department of Mechanical Engineering K. N. Toosi University of Technology Tehran 19991‐43344 Iran; ^2^ Department of Mechanical Engineering Technical University of Denmark 2800 Kongens Lyngby Denmark

**Keywords:** crossflow wind turbines, multiobjective optimization, power coefficient, smart building energy systems, technoeconomic feasibility studies

## Abstract

This work reports a technical, economic, and environmental investigation of the possibility of using a recently developed smallscale crossflow wind turbine (CFWT) to supply the energy demand of buildings for different integration scenarios. For this purpose, three CFWT‐assisted building energy system configurations with heat pumps, with and without batteries, and two‐way interaction with the local grid in two residential building models in Iran and Germany are investigated. Triobjective optimization with a Nondominated Sorting Genetic Algorithm (NSGA‐II) is performed for finding the optimal configuration of the energy system in different configurations. For economic assessment, the Capital Budgeting Analysis method is used with four indicators, namely, payback period (PP), net present value (NPV), internal rate of return (IRR), and profitability index (PI). The results show that due to different energy market regulations and prices, different integration scenarios and system configurations can outperform others in Germany and Iran. Overall, due to the exchange rate instability and low energy tariff in Iran, in order for the project to be feasible, either the CFWT cost must fall to below 30% of its current cost or the local electricity price should increase significantly to get a Levelized cost of energy of as low as 0.6 $ kWh^−1^.

## Graphical abstract

Small‐scale wind turbines have tremendous potential as a sustainable and pollution‐free energy solution. This work conducts a technical, economic, and environmental investigation as well as multiobjective optimization of crossflow wind turbines and heat pumps to supply the energy demand for the household sector in Iran and Germany. The effect of capital expenditure, weather conditions, and energy tariffs are discussed.

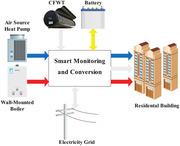

## 1 Introduction

The limited resources of fossil fuels and their environmental effects have seriously necessitated taking a pivot in the energy sources to be sustainable and pollution‐free.^[^ ^1^ ^]^ In this regard, several agreements, such as the Montreal Protocol 1987, Kyoto Protocol 1997, and Paris Climate Agreement 2015, have been established to restrict the use of ozone‐depleting substances and reduce greenhouse gas (GHG) emissions.^[^ ^2^ ^]^ Buildings consume about 40% of global energy and are responsible for approximately one‐third of the total world's GHG emissions.^[^ ^3^ ^]^ Previous research shows that proven and commercially available technologies can reduce buildings’ energy consumption by 30–80%.^[^ ^4^ ^]^ One of the concepts that could be much helpful for this is renewable‐based smart building energy systems with two‐way interaction with the energy grids.^[^ ^5^ ^]^ Among all renewable energy sources, abundant and affordable wind energy has been used by humankind for centuries, and small scale wind turbines (SSWTs), such as CFWTs, have grown and developed rapidly in the past few years.^[^ ^6^ ^]^ The most notable features of CFWTs are high starting torque at low wind speed, self‐starting capability, and simplicity of installation and structure, which together make them a suitable option for use in the urban environment.^[^ ^7^, ^8^ ^]^

Many researchers have already studied the techno‐economic aspects of renewable‐based smart building energy systems in Iran. Fazelpour et al.^[^ ^9^ ^]^ investigated the feasibility of supplying a household electrical demand on Qeshm Island. After simulating and optimizing several combinations, it was concluded that the wind turbine (WT) and photovoltaic (PV) hybrid system with battery storage is the most cost‐effective system, with a cost of energy (COE) of 0.604 $ kWh^−1^ and a total net present cost (NPC) of 30700 $ (based on 2015 US$). Baneshi and Hadianfard^[^ ^10^ ^]^ analyzed a WT‐PV‐diesel‐battery system for large nonresidential consumers with an average daily load demand of 9911 kWh in Shiraz. They presented the optimal hybrid system characteristics for off‐grid and on‐grid with the COE and renewable fraction values. Qolipour et al.^[^ ^11^ ^]^ ranked six regions in Ardabil province in terms of generated electricity, depreciation values, and total income and net cost for the construction of household WTs with a generator and batteries by a Nonfractional DEA‐BSC‐Game theory hybrid approach. Mohammadi et al.^[^ ^12^ ^]^ proposed a 100% renewable energy system for a residential single‐family house in Hesarek, Tehran. Their final optimal solution with minimizing the cost function and the reliability constraint consists of a 2‐kW WT, 4‐kW PV, 4‐kW inverter, and six battery strings. This scenario has NPC of 20527 $, a capacity shortage of 1.1%, and an unmet electricity load of 0.9%. Akbari et al.^[^ ^13^ ^]^ simulated the same hybrid system without batteries in Khvaf town, Razavi Khorasan Province. They concluded that the optimal design providing 93% of the electricity includes 13‐kW PV, 10‐kW inverter, and four horizontal axis wind turbines (HAWT) of 100 kW in on‐grid mode with an initial investment of 440 000 $ has a 4‐year payback period. For a residential complex in Tehran, Ashrafi Gudarzi et al.^[^ ^14^ ^]^ studied a WT‐PV‐battery hybrid system; additionally, Abbaszadeh et al.^[^ ^15^ ^]^ researched a WT‐PV‐gas generator system. Each separately indicated that hybrid systems instead of diesel systems could significantly reduce GHG emissions while achieving cost savings. Farahi and Fazelpour^[^ ^16^ ^]^ analyzed the WT‐PV‐battery‐diesel generator system for commercial, public, and residential buildings in Tehran, Kish, and Binaloud. Moreover, they did a sensitivity analysis on fuel cost, thereby announced Kish is the best region for all buildings. Residential buildings in Kish with a 60‐kW PV, a 2.5‐kW WT, 480‐kW diesel generator, and 16 batteries have a total COE of 0.250 $ kWh^−1^ and NPC of 15511300 $. The common features of these researches are that they all used three‐bladed HAWTs, and their renewable energy system was a hybrid system with batteries or, even in some cases, a gas generator. The disadvantages of these researches are the pollution caused by generators, the high initial investment cost, and the large space required for installing PV panels, WTs, batteries, and other equipment regardless of the maximum available space in residential buildings in Iran.

SSWTs have become increasingly popular in windy areas; however, few studies have focused on them in buildings worldwide. Rodriguez‐Hernandez et al.^[^ ^17^ ^]^ evaluated 28 SSWT models at 18 locations in the Valley of Mexico Metropolitan area to estimate annual energy production. Borunda et al.^[^ ^18^ ^]^ studied 5‐kW and 10‐kW SSWT to supply the residential demand in different Mexican cities with Intelligent Bayesian decision‐making. Ibrik^[^ ^19^ ^]^ analyzed 10, 100, and 220 kW SSWTs at eight sites in West Bank cities. Using a small‐scale HAWT, geothermal heat pump, and preheating with solar energy, Ozgener^[^ ^20^ ^]^ experimentally heated and cooled a greenhouse in Izmir, Turkey. He concluded that the WT alone can provide 3.1% of the total annual electricity consumption of the greenhouse (3568 kWh) or 12.5% of the total annual electricity consumption of the secondary water pumping, brine pumping, and fan coil (892 kWh). Mahmuddin et al.^[^ ^21^ ^]^ employed the Capital Budgeting Analysis method for a 300‐watt HAWT in the South Sulawesi coastal area, Indonesia, and determined the project is not feasible for wind speed below 7 m/s. Abdelrahim et al.^[^ ^22^ ^]^ examined and optimized wind‐driven charging stations for electric vehicles in high‐rise buildings in Malacca, Malaysia. The findings show that a 5‐kW RC‐5K‐A SSWT can generate 214 272 kWh yr^−1^ at the cost of 0.081 $ kWh^−1^, confirming the future feasibility of WT as a source of energy‐infrastructure support. Rahimi and Thaghafi^[^ ^23^ ^]^ studied the 5.5‐kW HAWT in the on‐grid mode in Borujerd, Iran and acknowledged that the PP is 14 years, although Shahbazi et al.^[^ ^24^ ^]^ declared the project with 5‐ and 10‐kW Hummer at the height of 10 m in a greenhouse in Robat Karim, Tehran (the same country as the Ref.[23#23]), requires government support to become feasible. All the SSWTs considered in the above investigations globally were small‐scale HAWTs. There are also two studies addressing the techno‐economic feasibility of small‐scale vertical axis wind turbines (VAWTs) in Iran, one analyzing a 2‐kW Savonius in Isfahan^[^ ^25^ ^]^ and another a hybrid Savonius‐Darrieus in Marvast, Yazd.^[^ ^26^ ^]^

One of the emerging types of SSWTs for building applications is CFWTs, on which there are not that many pieces of research. Indeed, although the scientific literature on smart energy buildings is exceptionally rich in terms of CFWTs, it suffers from several important gaps. First, after so many extensive studies focused on the performance and efficiency enhancement of CFWT, there is no research on its techno‐economic feasibility in the world. Second, no complete and comprehensive assessment has been conducted on small‐scale household WTs with/without batteries in on‐grid/off‐grid mode. The first and the second gaps can also be concluded from **Table** **1**. Three, the high investment costs of renewable‐based building energy systems, a substantial part of which is related to batteries, have discouraged building owners from implementing and installing them in their buildings; which could be addressed by studies in this class, the possibility of two‐way interaction of buildings with the electricity grid, and the possibility of multi‐generating by the WT employing heat pump units in the building. This work aims to provide a feasible and reliable path by employing all the above‐mentioned gaps toward developing CFWT‐integrated smart building energy systems. By considering this purpose, this study proposes a smart novel configuration integrated with the CFWT and air source heat pump having a two‐way interaction with the power grid. It helps the building owners to supply a part of their energy demands and compensate for building energy bills by selling their extra production electricity to the networks. Apart from this, two other CFWT‐based smart building energy systems equipped with batteries and heat pumps in on‐grid mode are presented. To get a better insight into the superiority and advantages of each configuration in different climates and countries, they are compared from technical, environmental, and economic aspects of different regions of Iran and also Germany as the case studies. Multiobjective optimization based on the Nondominated Sorting Genetic Algorithm (NSGA‐II) approach is applied to the configurations to determine optimal operating conditions, and various economic indices are employed for the economic analyses.

**Table 1:** Literature review (Bat = Battery, Gen = Generator, GHP = Geothermal heat pump)

Authors	Year	Location	Study area	Type of building	Design					Type of WT	Grid
					PV	WT	Bat	Gen	GHP		
Fazelpour et al.^[^ ^9^ ^]^	2015	Iran	Qeshm Island	Household	*	*	*			HAWT	Off‐grid
Baneshi and Hadianfard^[^ ^10^ ^]^	2016	Iran	Shiraz	Nonresidential consumers	*	*	*	*		HAWT	Off‐grid on‐grid
Qolipour et al.^[^ ^11^ ^]^	2016	Iran	Ardabil province	Household		*	*	*		HAWT	Off‐grid
Mohammadi et al.^[^ ^12^ ^]^	2018	Iran	Hesarek	Household	*	*	*			HAWT	Off‐grid
Akbari et al.^[^ ^13^ ^]^	2018	Iran	Khvaf	–	*	*				HAWT	On‐grid
Ashrafi Gudarzi et al.^[^ ^14^ ^]^	2019	Iran	Tehran	Residential complex	*	*	*			HAWT	Off‐grid
Abbaszadeh et al.^[^ ^15^ ^]^	2020	Iran	Tehran	Residential complex	*	*		*		HAWT	Off‐grid
Farahi and Fazelpour^[^ ^16^ ^]^	2018	Iran	Tehran, Kish, and Binaloud	Residential, public, and commercial buildings	*	*	*	*		HAWT	Off‐grid
Rodriguez‐Hernandez et al.^[^ ^17^ ^]^	2019	Mexico	Valley of mexico metropolitan	–		*				SSWT	On‐grid
Borunda et al.^[^ ^18^ ^]^	2020	Mexico	Whole country	Residential		*				SSWT	–
Ibrik^[^ ^19^ ^]^	2019	Palestine	West Bank	Several households		*				SS‐HAWT	Off‐grid
Ozgener^[^ ^20^ ^]^	2010	Turkey	Izmir	Greenhouse	*	*	*		*	SS‐HAWT	Off‐grid
Mahmuddin et al.^[^ ^21^ ^]^	2021	Indonesia	South Sulawesi	–		*				SS‐HAWT	–
Abdelrahim et al.^[^ ^22^ ^]^	2022	Malaysia	Malacca	High‐rise Building		*	*			SS‐HAWT	Off‐grid
Rahimi and Thaghafi^[^ ^23^ ^]^	2006	Iran	Borujerd	Commercial Building		*				SS‐HAWT	On‐grid
Shahbazi et al.^[^ ^24^ ^]^	2017	Iran	Robat Karim	Greenhouse		*	*			SS‐HAWT	Off‐grid
Mehrani et al.^[^ ^25^ ^]^	2014	Iran	Isfahan	Residential complex		*				VAWT (Savonius)	–
Kouravand^[^ ^26^ ^]^	2018	Iran	Marvast	Household		*				VAWT (hybrid Savonius‐Darrieus)	–

## 2 Proposed Building Energy System

**Figure 1:**
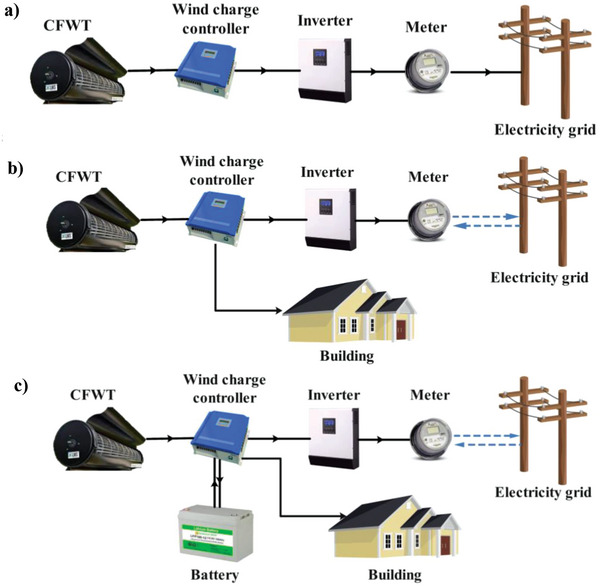
Schematic diagrams of a) configuration 1, b) configuration 2, and c) configuration 3.

**Figure** **1** demonstrates the schematic diagram of each analyzed smart building system. Systems consist of CFWT, wind charge controller, inverter, and battery. They differ based on grid interaction and battery presence or absence. A detailed description of each configuration is provided below:

a) Configuration 1 – with CFWTs, wind charge controller, and inverter without connection to the building, as shown in Figure 1a. This configuration is popular for on‐grid areas in Iran. According to **Table** **2**, the electricity tariff for the household sector is much cheaper than the wind feed‐in tariff.^[^ ^27^, ^28^ ^]^ Therefore, buying electricity from the grid and selling generated CFWT power to the grid is more economical. Unlike the electricity tariff in the household sector, which varies by hour, consumption level, and climate zone, the wind feed‐in tariff (FiT) is always fixed during a year.

**Table 2:** Electricity price in 2021

Parameter	Electricity price	Price changes	Conditions
Average electricity tariff for the household in Iran^[^ ^27^ ^]^	0.0024 $ kWh^−1^	+ 0.00012 $ kWh^−1^ annually (in the last 11 years)	Low consumption subscribers in temperate areas
Wind FiT in Iran^[^ ^28^, ^29^, ^30^, ^31^, ^32^ ^]^	0.0415 $ kWh^−1^	+ %13.17 annually (in the last seven years)	Capacity 1 MW and less
Average electricity tariff for the household in Germany^[^ ^33^, ^34^ ^]^	0.339 $ kWh^−1^	+ 0.0069 $ kWh^−1^ annually (in the last 11 years)	–
Wind FiT in Germany^[^ ^35^ ^]^	0.0588–0.142 $ kWh^−1^	–	Price changes depending on the location and network demands.

b) Configuration 2 – with CFWTs, wind charge controller, and inverter, as illustrated in Figure 1b. Depending on the building's electricity demand assessment, the smart controller decides whether to sell electricity to the grid or supply the building's demand. Otherwise, when there is not any electricity demand, the extra generated electricity sells to the grid.

c) Configuration 3 – with CFWTs, wind charge controller, inverter, and battery, as presented in Figure 1c. In this configuration, the priority is to supply building electricity through the grid (a better decision based on the tariffs in Iran). Additionally, the battery is applied to supply the building's required electricity when the grid power is cut off, or the building demand is high during peak tariff hours. In addition, it has a two‐way interaction with the electricity grid to sell extra electricity and purchase from the grid.

## 3 Experimental Section

The demand energy profile of buildings for one year is calculated by Carrier HAP 4.90 software. Afterward, to perform economic and environmental assessment, the modeling of the proposed smart building systems is analyzed via MATLAB software with a time resolution of 1 hour. Finally, the tri‐objective optimization using the NSGA‐II algorithm approach is applied to the scenarios to find the best operating condition from technical, economic, and environmental facets.

### 3.1 The Case Studies

**Figure 2:**
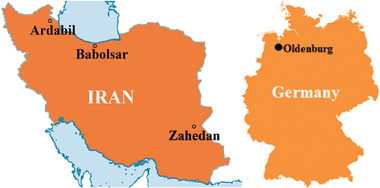
Location of the case study cities in Iran and Germany.

The cities of Ardabil, Zahedan, and Babolsar, located in Iran based on **Figure** **2**, were considered case studies of this work. According to Köppen–Geiger climate classification, they are in hemi boreal, arid, and humid subtropical climates, respectively.^[^ ^36^ ^]^ Also, based on the climate and wind characteristics similarity, Oldenburg city in Germany was chosen for comparison and illustrated in Figure 2. Two residential buildings are contemplated: a 160 m^2^ house on a field of 14 m × 11.5 m and a 4‐story apartment with two 65 m^2^ units. The house is simulated in low‐consumption and high‐consumption patterns. Residents of the low‐consumption house have proper timing and saving in using electrical energy. The prerequisite data for calculating the cooling, heating, and electrical profiles with Carrier HAP software, contained the characteristic of the buildings, local weather information, comfort standards, and hot water demand profiles. Cooling and heating systems were selected based on the city climate and available equipment.

### 3.2 Economic Analysis

The total capital expenditure during the lifetime (20 years) was calculated and compared with the grid electricity costs, a conventional method in Iran, to evaluate the proposed configurations from an economic perspective. The total cost (*C*_tot_) is the sum of all component costs over the system lifetime. The cost of each component (***C***_***k***_) equals the sum of the initial cost ($C_{k}^{\text{IC}}$) and operational and maintenance costs ($C_{k}^{\text{OM}}$) as follows

(1) $$\begin{array}{c} C_{\text{tot}}=\sum_{k=1}^{n}C_{k} \end{array}$$

(2) $$\begin{array}{c} C_{k}=C_{k}^{\text{IC}}+C_{k}^{\text{OM}}=C_{k}^{\text{IC}}\left(1+\gamma \right) \end{array}$$

Here the annual maintenance cost coefficient (γ) is the factor related $C_{k}^{\text{OM}}$ to the $C_{k}^{\text{IC}}$. **Table** **3** shows the costs of each component to accomplish the economic evaluation.

**Table 3:** Purchased cost of each component^[^ ^37^, ^38^, ^39^ ^]^

Component	Model or size	Lifetime	$C_{k}^{\text{CI}}[\$)]$	γ
Wind turbine	0.1, 0.3, and 0.5 kW	20 yr	$\begin{array}{c} C_{\text{WT}}=c_{1}\times CAP_{\text{WT}}\times N_{\text{WT}}\\ c_{1}=5836\$\;{\text{kWh}}^{-1}\\ CAP_{\text{WT}}=0.5\;\text{kWh} \end{array}$	0.02
Inverter	5 kW – single phase	10 yr	$\begin{array}{c} C_{\text{Inv}}=c_{2}\times CAP_{\text{Inv}}\\ c_{2}=120\$\;{\text{kWh}}^{-1}\\ CAP_{\text{inv}}=CAP_{\text{WT}}\times N_{\text{WT}} \end{array}$	–
Battery	1.2 kWh – deep cycle gel	12 yr	$\begin{array}{c} C_{\text{Bat}}=c_{3}\times CAP_{\text{Bat}}\\ c_{3}=132.5\$\;{\text{kWh}}^{-1} \end{array}$	0.002
Wind charge controller	12 V/24 V –300 W/600 W	10 yr	*C*_Ch_ = 31$	–
Wiring, foundation, and installation fee	–	–	*C*_Fee_ = 0.1 × (*Z*_WT_ +*Z*_Inv_ +*Z*_Ch_ + Z_Bat_)	–

The net present value over the lifetime is defined as below

(3) $$\begin{array}{c} \text{NPV}=\sum_{i=1}^{n}\frac{AC_{i}^{\text{SP}}-AC_{i}^{\text{wind}}-C_{\text{tot},i}^{\text{OM}}}{{\left(1+x\right)}^{i}}-C_{\text{tot}}^{\text{IC}} \end{array}$$

where *x* is the discount rate and $C_{\text{tot},i}^{\text{OM}}$ is the total operational and maintenance costs for the ith year. Moreover, the IRR describes an investment return rate as the percentage at which the NPV value is zero. *AC* ^wind^ is the annual cost of wind‐based and *AC* ^SP^ is the annual cost of separation production systems, which are written as^[^ ^40^ ^]^

(4) $$\begin{array}{c} AC^{\text{wind}}=\left({\dot{E}}_{\text{Bought}}-{\dot{E}}_{\text{Sold}}\right)\times c_{\text{electricity}} \end{array}$$

(5) $$\begin{array}{c} AC^{\text{SP}}={\dot{E}}_{\text{Demand}}\times c_{\text{electricity}} \end{array}$$

*c*_electricity_ is the electricity price based on Table 2 and ${\dot{E}}_{\text{Demand}}$is the total electricity demand for the whole year. Another ranking investment index where the value of 1 indicates the break‐even point is PI

(6) $$\begin{array}{c} \text{PI}=\frac{\text{NPV}+C_{\text{tot}}^{\text{IC}}}{C_{\text{tot}}^{\text{CI}}} \end{array}$$

In the levelized cost of energy (LCoE), the cost of producing 1 kWh of electricity is calculated by dividing the present value of all the costs by the CFWT electricity production in the project lifetime^[^ ^41^ ^]^

(7) $$\begin{array}{c} \text{LCoE}=\frac{\sum_{i=1}^{n}\frac{C_{i}^{CI}+C_{i}^{OM}}{{\left(1+x\right)}^{i}}}{\sum_{i=1}^{n}\frac{{\dot{E}}_{\text{wind},i}}{{\left(1+x\right)}^{i}}} \end{array}$$

${\dot{E}}_{\text{wind,i}}$ is the CFWT electricity production in the ith year. Another important economic indicator is the PP, defined as the number of years necessary to compensate for the initial costs

(8) $$\begin{array}{c} \text{PP}=\frac{C_{\text{tot}}}{AC^{\text{SP}}-AC^{\text{Wind}}} \end{array}$$

Furthermore, dependency on the grid (DoG) indicates the percentage of the annual energy requirement provided by purchasing from the grid, as follows

(9) $$\begin{array}{c} \text{DoG}=100\times \frac{\sum {\dot{E}}_{\text{Bought}}}{\sum {\dot{E}}_{\text{Demand}}} \end{array}$$

### 3.3 Environmental Analysis

Due to the increase in global environmental pollution, especially the worrying emission of GHG and its dangerous effect on rising temperature and changing wildlife and human life worldwide, environmental analysis has become more significant than ever. Here, the carbon dioxide emission reduction rate (CDERR) is calculated to compare the proposed integrated building CFWT configurations and demonstrate their superiority over the conventional method based on Equation (10)

(10) $$\begin{array}{c} \text{CDERR}=\frac{CDE^{\text{SP}}-CDE^{\text{wind}}}{CDE^{\text{SP}}}\times 100 \end{array}$$

(11) $$\begin{array}{c} CDE^{\text{wind}}=\left({\dot{E}}_{\text{Bought}}-{\dot{E}}_{\text{Sold}}\right)\times \delta \end{array}$$

(12) $$\begin{array}{c} CDE^{\text{SP}}={\dot{E}}_{\text{Demand}}\times \delta \end{array}$$

where *CDE* ^wind^ is the carbon dioxide emissions of wind‐based and *CDE* ^SP^ is the separation production systems in kg. The carbon dioxide emission coefficient (δ) based on the energy balance sheet published by the Ministry of Energy is 0.645 kgCo_2_ kWh^−1^. As a result of electricity generation from CFWT, the non‐emission of Co_2_ can be calculated as follows^[^ ^42^ ^]^

(13) $$\begin{array}{c} Ton\;{\text{Co}}_{2}=\frac{{\dot{E}}_{\text{wind}}\times \delta }{1000} \end{array}$$

### 3.4 Multiobjective Optimization

The purpose of multiobjective optimization is to improve several conflicting performance indexes simultaneously according to the limitations and constraints of the problem. In many engineering fields, there are conflicts between objective functions, such as cost, time, and performance. Thus, finding a balance point between them is essential to maintaining system performance while reducing cost and time. The Genetic Algorithm (GA) is an efficient method to solve problems that are usually intractable or time‐consuming to solve by other methods.^[^ ^43^, ^44^ ^]^ Compared to other methods, it has the fastest computational speed and the highest number of best objective functions.^[^ ^45^ ^]^

Further, GA was not getting stuck in local optimal points. In the present study, tri‐objective optimization using the Nondominated Sorting Genetic Algorithm (NSGA‐II) approach^[^ ^46^ ^]^ is implemented to find the best decision variables for each configuration. MATLAB software was applied to maximize NPV (Equation (3)) and CDERR (Equation (10)) as fitness functions while simultaneously minimizing dependency on the grid (Equation (9)) as a cost function. Variable parameters for optimization were battery capacity and number of CFWT with a domain of 0 kWh < *CAP_Bat_* < 10 kWh and 0 < *N_WT_* < 10, respectively.

After the algorithm termination, the Pareto front is obtained for each generation. Choosing an answer among a set of answers (Pareto front) is difficult for most decision‐makers. However, researchers have introduced different criteria; for example, the CS point is based on the nearest distance to the ideal point, and the ES point is the intersection of the line passing through Threat point c with an angle of 45° with the Pareto front.^[^ ^47^ ^]^

## 4 Results and Discussion

The technical, economic, and environmental assessments of the proposed smart energy home systems are surveyed by using MATLAB software. A prerequisite information required to perform the simulation is the house energy demand profile, calculated with Carrier HAP, a powerful and comprehensive tool for designing HVAC systems and modeling annual energy performance and energy costs. Time‐dependent, hourly and monthly charts are first extracted to detect the best configuration. Then, a comparative parametric analysis is conducted to evaluate the effect of the main parameters on the technical, economic, and environmental indicators. Finally, considering NPV, CDERR, and dependency on the grid as objective functions, multiobjective optimization based on the NSGA‐II approach is applied for the best city considering all aspects.

### 4.1 Parametric Study of the Configurations

**Figure 3:**
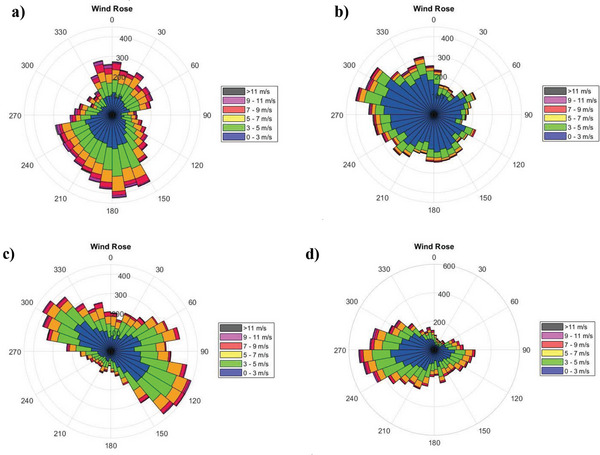
The wind rose for a) Ardabil, b) Babolsar, c) Zahedan, and d) Oldenburg.

**Figure 4:**
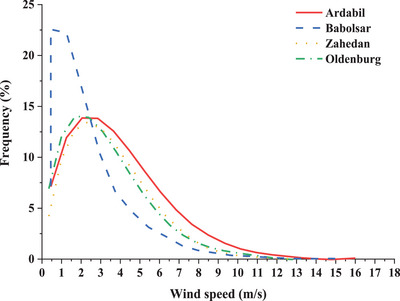
Frequency of wind speed in different cities.

**Figure 5:**
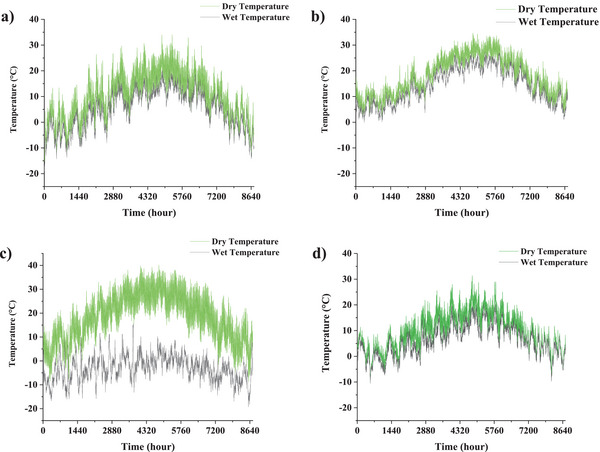
Variation of the ambient temperature of a) Ardabil, b) Babolsar, c) Zahedan, and d) Oldenburg for the whole year.

The desired location for installing the CFWT must have suitable wind speed and direction conditions. The wind direction should not have sudden fluctuations, the average wind speed should be high, and the highest prevailing wind speed should be in one direction. The best way to display and analyze these features is through Wind rose and wind speed frequency diagrams, as shown in **Figures** **3** and **4**. The dispersion of the wind direction in Babolsar from the southeast to the northwest makes the best installation option to be VAWT. The dominant wind direction is south to the southwest, with an average wind angle of 200° in Ardabil. If the turbine blades are placed at this angle, they can absorb maximum southwest wind potential from the front and northeast from behind the turbine. This angle is 120° for Zahedan and 265° for Oldenburg. In Figure 4, the average annual wind speed data at each city is classified by the Sturges’ rule. The wind speed in Ardabil is 3.87 ± 2.46 m s^−1^ (3.87 is mean and 2.46 is variance), Zahedan is 3.48 ± 2.05 m s^−1^, Oldenburg is 3.31 ± 2.19 m s^−1^, and Babolsar is 1.139 ± 1.2 m s^−1^. Thus, the occurrence probability of winds exceeding 1.2 m s^−1^ is higher in Ardabil, Zahedan, and Oldenburg than in Babolsar. Another weather information, the hourly variation of ambient temperature, is shown in **Figure** **5**, where a noteworthy point in this figure is a significant difference, about 16.7 C°, between the day and night temperatures of Zahedan because of the dry weather.

**Figure 6:**
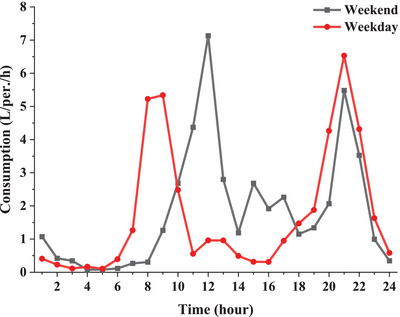
Diagram of average hourly hot water consumption per person on working days and holidays.^[^ ^48^ ^]^

**Figure 7:**
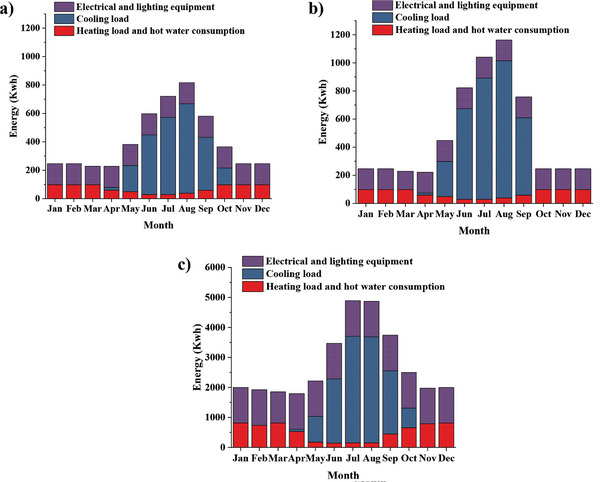
Monthly heating, cooling, and electricity demand of a) low consumption house, b) high consumption house, and c) apartment.

**Figure 8:**
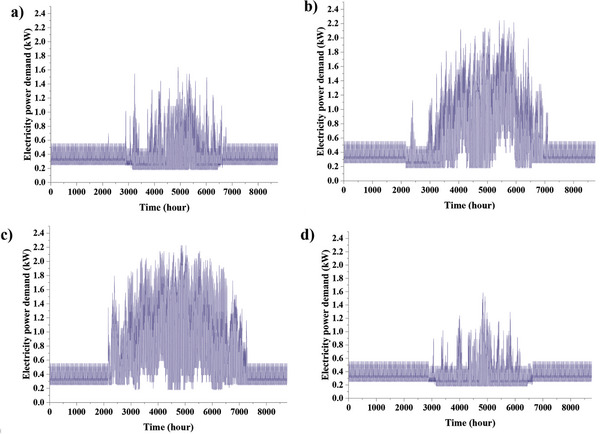
The variation of hourly electricity demand in a) Ardabil, b) Babolsar, c) Zahedan, and d) Oldenburg.

**Figure 9:**
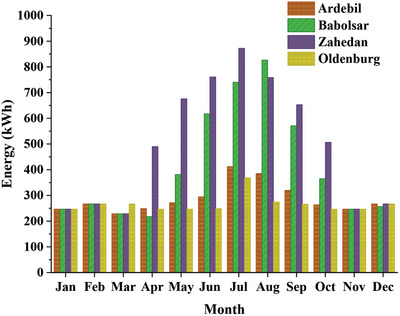
The variation of monthly electricity demand in 4 selected cities.

The average hourly hot water consumption profile for a person living in a family of four on working days and holidays is illustrated in **Figure** **6**.^[^ ^48^ ^]^ The power consumption characteristics of electrical equipment are presented in **Table** **4**. These data have been used to simulate the demand profile of heating, cooling, and electricity in buildings, as presented in **Figure** **7**. The cooling device is an evaporative cooler in hot and dry cities or a heat pump in other cities. Also, a Wall‐hung gas boiler is used for heating and hot water supply to the building. The electrical energy used by the cooling and heating equipment is added to the electricity demand profile. **Figures** **8** and **9** show the hourly and monthly electricity demand in the low‐consumption house for four cities. It can be seen that Zahedan, which has a hot and dry summer climate, has the largest electricity demand, followed by Babolsar.

**Table 4:** The list of appliances with their nominal power, standby power, daily frequency, and operational cycle duration^[^ ^49^, ^50^ ^]^

Application	Nominal power [W]	Standby power [W]	Duration [h]	Daily frequency
Refrigerator	110	8.1	0.2	40.5
Freezer	110	8.1	0.2	40.5
Microwave oven	1500	0	0.0833	4
Coffee maker	1000	0	0.1	0.7
Range oven	1050	8	0.2	0.38
Hair dryer	1600	0	0.1	1.2
TV	105	4	1	0.12
Computer	110	2.5	1	2.3
Air conditioning	220	0	2	1.3
Other occasional loads	1000	0	1	0.66
Clothes washer	1200	0	1	0.41
Lighting	120	0	0.5	12
Wi‐Fi adaptor	7	0	1	1.1
Vacuum	1500	0	0.1	0.41
Laptop	27	2	0.4	4

**Figure 10:**
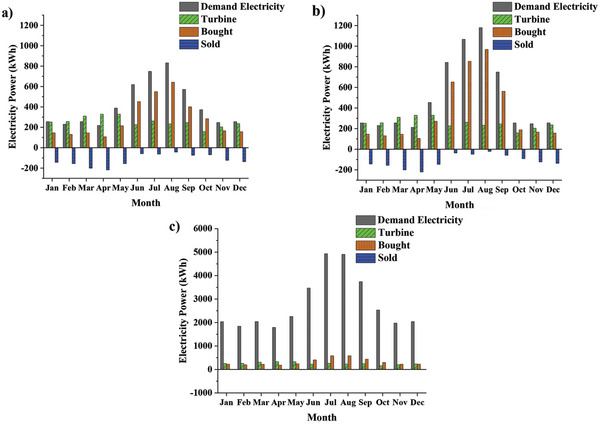
Monthly variation of the generated CFWT power, electricity demand, and bought/sold electricity from/to grid in a) low consumption house, b) high consumption house, and c) apartment.

According to the turbine dimensions in **Table** **5** and the apartment and house rooftop area, installing a maximum of ten 0.5‐kW CFWTs is possible. **Figure** **10** shows the monthly variation of the produced CFWT power, electricity demand, and electricity bought and sold in three building types in Babolsar with configuration 2. According to Figures 10a,b, monthly electricity costs are saved, and owners also receive a long‐lasting income from selling CFWT electricity. In the case of Figure 10c, the 8‐unit apartment consumed all the produced CFWT power, and there is nothing left to sell to the grid. Configuration 2 reduces energy costs by 39% to 69% in a low‐consumption house, 25–69% in a high‐consumption house, and 9% to 50% in each apartment unit, depending on the season.

**Table 5:** Characteristics of CFWTs^[^ ^51^ ^]^

Parameters	100LWS	300LWS	500LWS
Rotor diameter [mm]	180	355	355
Length [mm]	1200	1120	1120
Weight [kg]	13	25	28
Cut‐in wind speed [m s^−1^]	1.2	1.2	1.2
Expected power production in 3 m s^−1^ [kWh yr^−1^] {approximately}	160 {130}	610 {500}	960 {700}
Expected power production in 5 m s^−1^ [kWh yr^−1^] {approximately}	390 {310}	1100 {880}	1490 {1200}
Expected power production in 7 m s^−1^ [kWh yr^−1^]	450	1300	2100
Expected power production [kWh yr^−1^]	700 @9.6 m s^−1^	1900 @10.8 m s^−1^	3200 @11.4 m s^−1^
Rated power [W]	100	300	500
Capital cost	2918 $	2918 $	2918 $
Lifetime [yr]	20	20	20

**Figure 11:**
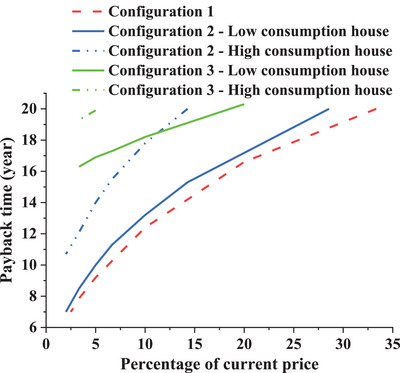
The variation of the PP in terms of the percentage of current price for each configuration and building in Babolsar.

**Figure** **11** shows the PP for each configuration and building in Babolsar. Due to the PP of the apartment being longer than the project lifetime, it cannot be displayed in this figure. Moreover, not only does configuration 1 has a shorter PP, but also it has a higher IRR and NPV that induce more cost‐effectiveness. When the power consumption pattern of a house is observed, it reduces home electricity costs further and results in a significantly different PP between low‐ and high‐consumption houses. The usage of CFWT in the apartment is problematic because of the following reasons:

- Most buildings have unused rooftop space under high wind speed conditions, making WTs a suitable option. Nonetheless, Turbulent and eddy wind flows caused by adjacent trees and high‐rise buildings are critical subjects that must be studied before installing the WTs.
- Apartment residents face the challenges of operating the WT system, such as obtaining all owners’ consent before the integration, participating in the financial profits and losses, determining a person responsible for the WT system monitoring and maintenance, and understanding financial contracts and regulations.

**Figure 12:**
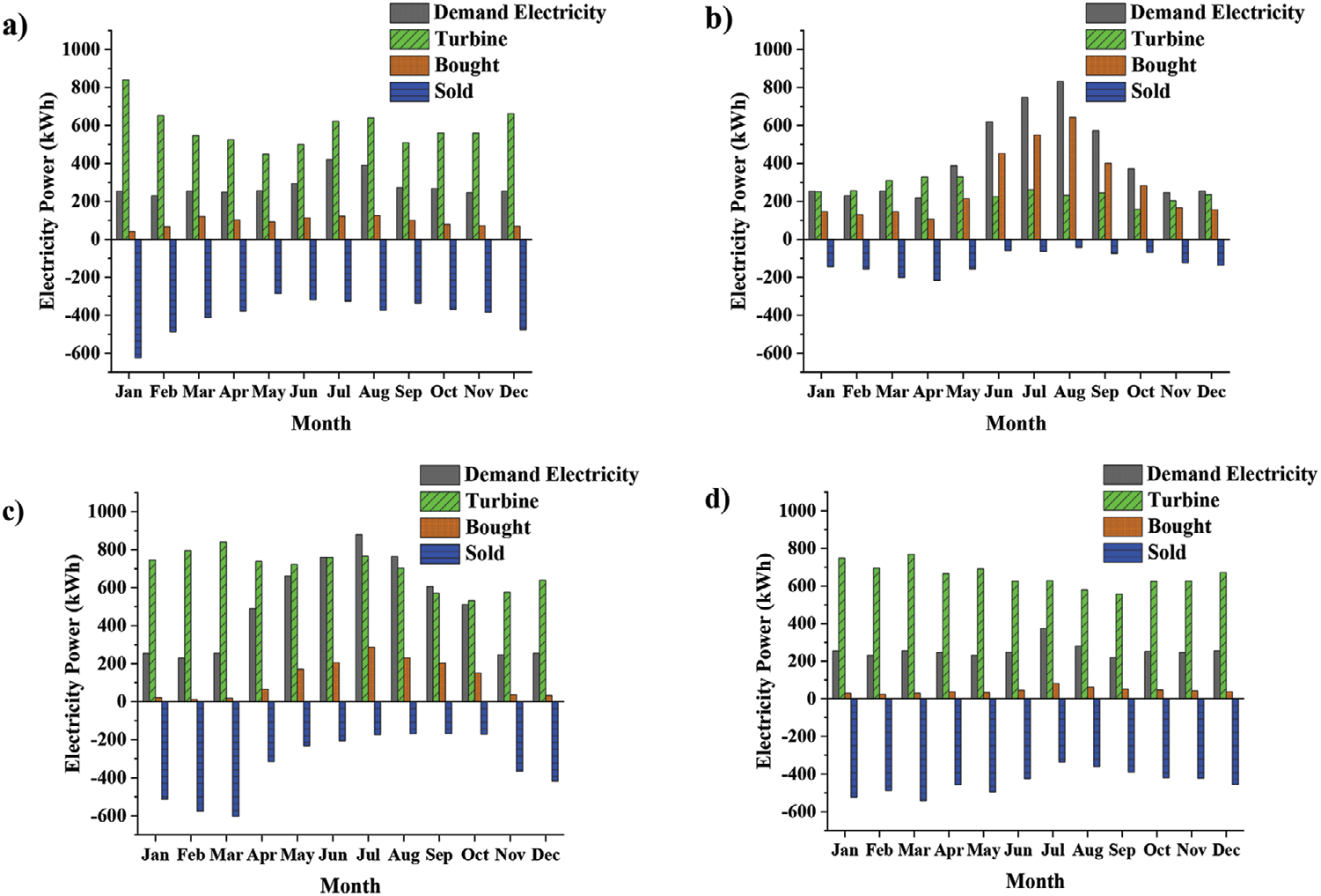
Monthly variation of the generated CFWT power, electricity demand, and bought/sold electricity from/to grid in a) Ardabil, b) Babolsar, c) Zahedan, and d) Oldenburg.

The effect of city and climate is investigated by considering building type, configuration, and turbine price as fixed parameters. For this purpose, CFWTs integrated with the house at the current price are considered for simulation. Referring to **Figure** **12**, the CFWT electricity production is high in fall and winter in Ardabil (average of 629 kWh at six months), which leads to the purchase of a small portion of electricity demand from the grid. The CFWTs in Zahedan produce an average of 300 kWh of electricity per month. However, due to the high electricity demand from April to October, a small amount of CFWT electricity is for sale, and even a part of the electricity demand must be purchased from the grid. The CFWTs in Oldenburg have uniform productions throughout the year, and electricity demand is less than the turbine production. All this has led to less buying from the grid and more selling. On the other hand, the turbine production in Babolsar is deficient because of the maximum of 329.56 kWh in May; therefore, more electricity is purchased from the grid, and it does not seem cost‐effective compared to other cities.

**Figure 13:**
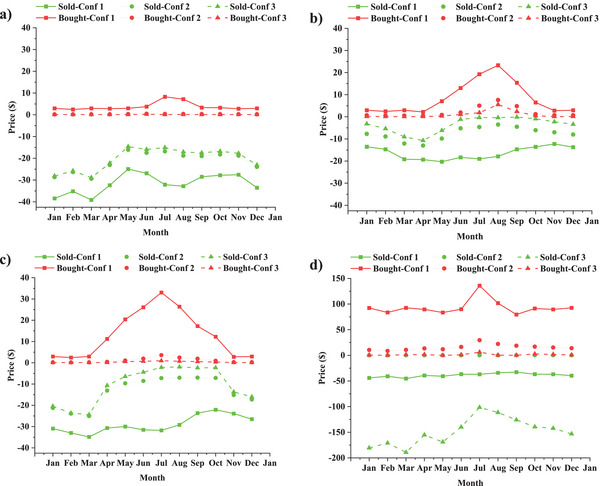
Monthly price variation of sold and bought electricity in three configurations in a) Ardabil, b) Babolsar, c) Zahedan, and d) Oldenburg.

**Figure** **13** delineates the monthly price of bought/sold electricity from/to the grid in each configuration, assuming that the number, price, and capacity of CFWTs in the house are constant. Based on this figure, in configuration 2, Ardabil and Zahedan pay a small amount for the electricity bills and earn profits from selling surplus turbine electricity. In Oldenburg, the electricity bill has decreased by an average of 83.6% per month; however, there is a negligible profit from selling surplus turbine electricity, a max of 1.54 $. Using configurations 2 and 3 has led to a drastic electricity bill reduction in Babolsar (87.31% and 92.21%) and Zahedan (94.43% and 97.17%). Likewise, the annual energy cost in Oldenburg decreased significantly by 83.6% in configuration 2 and 98.65% in configuration 3. A remarkable aspect of Oldenburg is that selling the electricity stored in batteries to the grid during periods of low demand can earn a high profit.

**Figure 14:**
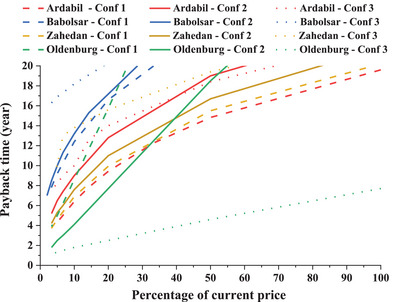
The variation of the PP in terms of the percentage of current price for three configurations and four cities.

**Figure 15:**
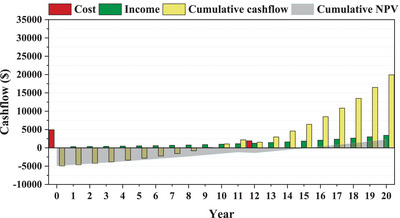
Cashflow analysis in Ardabil for configuration 3.

The PP in all configurations is depicted in **Figure** **14**, where the graphs of Ardabil, Zahedan, and Babolsar are logarithmic curves, and the graph of Oldenburg is linear due to the constant annual FiT. As mentioned, Ardabil experiences higher wind speeds and frequency than other cities. Subsequently, the CFWT power generation will be higher, and the PP will be smaller in all configurations. If a 5‐year PP in configuration 1 is desired, the CFWT price should reach 6.7%, 6%, and 5% of the current price in Ardabil, Zahedan, and Oldenburg, respectively. The graphs of configuration 3 for Ardabil and Zahedan slightly deviated from their normal behavior. This deviation is the PP time jump that occurred due to battery replacement. According to **Figure** **15**, in the 12th year, the battery replacement costs will result in a negative net cash flow and a sudden bad jump in economic metrics, including the PP.

According to **Table** **6**, for the cities of Iran, the PP in configuration 1 is the smallest; however, in Oldenburg, configuration 3 has the smallest PP. In other words, configuration 3 with the CFWT current price has a PP of less than eight years, and it is the best configuration in Oldenburg. Meanwhile, configurations 1 and 2 will have a PP less than the system lifetime if the CFWT price decreases. The purchased expensive household electricity and sold cheap wind electricity are the reasons for the economic attractiveness of configuration 3 compared with the first one. On the other hand, the results in Iran are entirely different from Germany due to the cheap and available household electricity. For instance, only configuration 1 in Ardabil at the current price will have a PP of 19.6 years, while other indexes show a nonfeasible project (with NPV, IRR, and PI of −22 900, 0.32, and 0.3, respectively). The PP of other cities of Iran at the CFWT current price is more than the system lifetime, but its reduction depends on the devaluation of the CFWT. That means by reducing the CFWT price to 10% of the current price in all cities and configurations, the PP will be less than 14 years, except for configuration 3 in Babolsar. In short, it can be acknowledged that configuration 1 in Iran will be more economically profitable if the following two conditions are met:

1. Choosing cities with an average speed of more than 3 m s^−1^; and
2. A devaluation of CFWT from 29% to 10% of its current price.

**Table 6:** The PP indicator of the CFWT‐based system in three configurations and four cities

Turbine price	Current price			50% of current price			10% of current price		
Configuration	1	2	3	1	2	3	1	2	3
Ardabil	19.6	–	–	14.83	19	18.4	6.4	9	10
Babolsar	–	–	–	–	–	–	12.4	13.2	18.2
Zahedan	–	–	–	15.5	16.7	19.4	6.9	7.6	13.8
Oldenburg	–	–	7.7	–	18.5	4.6	8.6	4.1	1.8

Figure 15 shows the economic analysis criteria for Ardabil, including cost, income, cumulative cash flow, and cumulative NPV. 5‐kW total capacity CFWTs with a price reduction of up to 6.7% are used to plot this figure. What stands out from Figure 15 is that in the zeroth year, the year of the construction, the project is only costly and has no income. The cumulative cash flow is negative in the 8th year and positive in the 9th; thereby, the project reaches net profitability in the 9th year.

**Figure 16:**
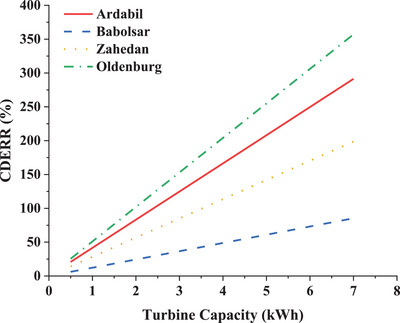
The variation of CDERR in configuration 2.

The reduction rate of carbon dioxide emissions is shown in **Figure** **16**. CDERR in the presence of the CFWT will be higher in any city with less electricity demand, less purchased from the grid, and more sold to the grid. As a result, the CDERR parameter increases in Babolsar, Zahedan, Ardabil, and Oldenburg, respectively. It should be mentioned that the pattern of consumption and type of electrical equipment in all cities is assumed to be the same to investigate the turbine performance.

Another proposed correction factor is the LCoE, calculated using the CFWT current price. Using configuration 1, the most economical configuration in Iran, the LCoE for the 5, 10, and 20 years PP is calculated in **Table** **7**. The number of 4 and 7 CFWTs are considered from the multiobjective optimization results, as presented in **Table** **8**. If the 10‐year PP is desired, the local electricity price should increase significantly to get the LCoE of 0.6 $.

**Table 7:** LCoE based on the PP of 5, 10, and 20 years for 4 and 7 turbines

Period	5 years		10 years		20 years	
	4 turbines	7 turbines	4 turbines	7 turbines	4 turbines	7 turbines
Ardabil	0.89	0.87	0.55	0.54	0.4	0.39
Babolsar	2.69	2.63	1.66	1.63	1.21	1.18
Zahedan	0.97	0.95	0.6	0.59	0.44	0.43

**Table 8:** Optimization results based on Pareto front diagrams

	Objective function/ Variable parameters	Configuration 2	Configuration 3
Variable parameters range	Number of turbines	0–10	0–10
	Battery capacity [kWh]	–	0–10
Ideal point	NPV [$]	2972.74	2724.25
	Dependency [%]	24.14	15.72
	CDERR [%]	142.41	142.5
CS point	NPV [$]	2320.13	1253
	Dependency [%]	38.42	28.38
	CDERR [%]	90	115
	Battery capacity [kWh]	–	0.964
	Number of turbines	7	8
ES point	NPV [$]	2588.86	1935.57
	Dependency [%]	58.56	51.03
	CDERR [%]	47.19	57.45
	Battery capacity [kWh]	–	0.97
	Number of turbines	4	4

### 4.2 Optimization Results

**Figure 17:**
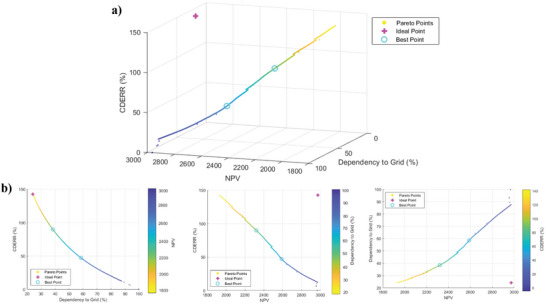
a) 3D Pareto front diagram and b) 2D Pareto front diagrams of NPV, CDERR, and grid dependency for configuration 2.

**Figure 18:**
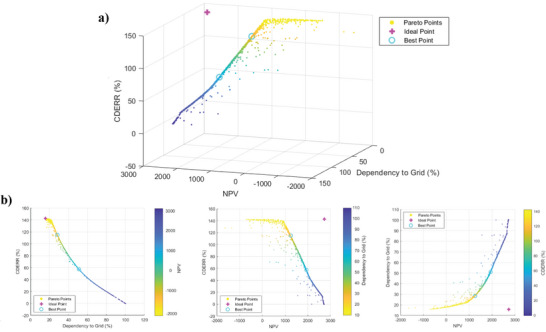
a) 3D Pareto front diagram and b) 2D Pareto front diagrams of NPV, CDERR, and grid dependency for configuration 3.

**Figure 19:**
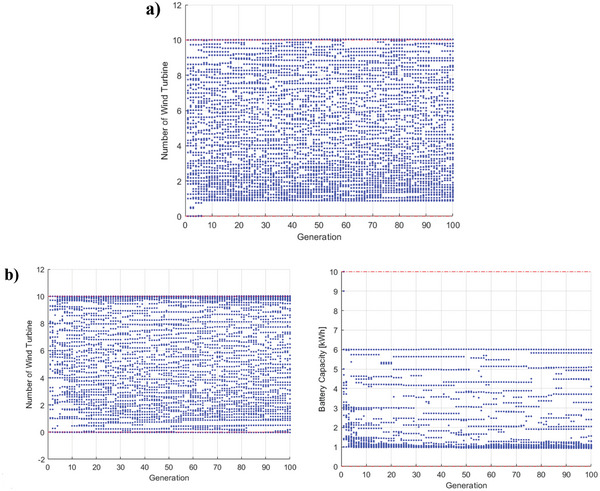
Scatter distribution of main variable parameters during successive generations for a) configuration 2, b) configuration 3.

As discussed, the conflict between objectives in configurations 2 and 3 reveals the importance of multiobjective optimization. Using a tri‐objective optimization approach based on the NSGA‐II, objective functions of NPV, CDERR, and grid dependency are optimized for Zahedan city by the devaluation of CFWT to 29% of the current price. Accordingly, the Pareto front diagram for all generations is shown in **Figures** **17** and **18**. The 2D diagrams are illustrated in Figures 17b and 18b to better comprehend the Pareto front diagram. CS and ES methods are used to calculate the best points shown along with the ideal point in these figures. The optimization results, including the optimal value of the decision parameters and the obtained objective functions, are listed in Table 8. According to the table, if maximizing NPV is the only goal, configuration 2, with an NPV of 2588.86 $, is the desired condition. However, the lowest grid dependency and the highest CDECRR occur in configuration 3 with 28.38% and 115%, respectively. In view of the fact that NPV is more important than the other two objective functions for assessing wind‐based building energy systems, configuration 2 seems to be more economic. As seen in Table 8, the optimum battery capacity value is about 1 kWh, and the number of turbines in configurations 2 and 3 is almost the same. The scatter distributions of variable parameters are illustrated in **Figure** **19** for a better image of the optimum range. From Figure 19a, it can be concluded that the optimal number of turbines is more than one. Referring to Figure 19b, since all the number of turbine optimal points have been distributed in the entire range from 0 to 10 with a concentration at the upper bound, it is not a sensitive parameter. However, the battery capacity is a sensitive parameter, and keeping it between 1 to 6 kWh leads to optimal technical and economic conditions simultaneously.

## 5 Conclusion

This work focused on smart building systems integrated with CFWTs to reach maximum energy performance, reliability, and cost‐effectiveness for residential buildings. The proposed smart systems have been simulated with/without batteries in on‐grid mode and two‐way interaction with the electricity grid to provide electricity and cooling for two typical residential buildings in selected cities of Iran and Germany. Comparisons and analyses have been made considering the weather conditions, tariffs, and energy regulations of both countries. After calculating the building demand energy profile, MATLAB software is used to simulate and compare all configurations from technoeconomic‐environmental aspects. Multiobjective optimization based on the NSGA‐II approach is applied to study the influence of variable parameters containing the number of turbines and battery capacity on the performance of configurations 2 and 3. These parameters are examined by evaluating their impact on the NPV, CDERR, and grid dependency. In summary, the substantial conclusions are drawn as follows:

- A low‐consumption house is the best building model for installing a CFWT in Iran. Configurations 1, 2, and 3 will be efficient, respectively.
- Although Zahedan and Babolsar have the highest electricity demand because of their hot and dry summer, configurations 2 and 3 have reduced Zahedan's electricity bill by 94.43% and 97.17%, and Babolsar's by 87.31% and 92.21%.
- Configuration 1 is the most cost‐effective and the best in Iran, which will increase the indicators if two conditions are met:
  1. Utilization in windy, moderate climate cities with an average wind speed of over 3 m s^−1^; and
  2. Devaluation of CFWT to 29% of the current price by allocating a subsidy or producing a similar model at a lower price.
- The best option from the economic aspect in Oldenburg is configuration 3. Due to its expensive household electricity tariff and cheap wind FiT, it varies from the best configuration in Iran.
- The PP less than the system lifetime in Iran is achieved by reducing CFWT price or changing LCoE. Thus, in Ardabil, Zahedan, and Babolsar, if the 10‐year PP for configuration 1 is taken into consideration, either the CFWT price should decrease to 21%, 20%, and 6% of the current price, or the local electricity price should increase significantly to get LCoE of 0.54 $, 0.59 $, and 1.63 $, respectively.
- Configurations 2 and 3 are currently not attractive in Iran, since the wind FiT is 17 times higher than the household electricity tariff.
- The tri‐objective optimization in Zahedan with the devaluation of CFWT to 29% of the current price indicates the NPV, grid dependence, and CDERR of seven turbines have values of 2330 $, 38.42%, and 90%, respectively, by choosing configuration 2. Furthermore, selecting configuration 3 changes objectives to 1253 $, 28.38%, and 115%, as well as variable parameters to eight turbines and 1 kWh battery capacity.
- The maximum NPV and CDECRR occurred in configuration 2 with 2588.86 $ and configuration 3 with 115%, respectively. In contrast, the minimum grid dependency happened in configuration 3, with 28.38%.
- Scatter distribution of variable parameters reveals that while the number of turbines is not a sensitive parameter, keeping the battery capacity at the lower bound leads to suitable performance/economic outcomes.

## Conflict of Interest

The authors declare no conflict of interest.

## Data Availability

The data that support the findings of this study are available from the corresponding author upon reasonable request.

## References

[1] A. Arabkoohsar , A. Behzadi , N. Nord , Energy Convers. Manage. 2021, 237, 114120.

[2] A. R. Razmi , A. Arabkoohsar , H. Nami , Energy 2020, 210, 118559.

[3] M. Azaza , D. Eriksson , F. Wallin , Energy Convers. Manage. 2020, 213, 112807.

[4] UNEP, Energy efficiency for buildings, France [Online] , https://www.euenergycentre.org/images/unepinfosheet-eebuildings.pdf.

[5] A. Arabkoohsar , A. Behzadi , A. S. Alsagri , Energy Convers. Manage. 2021, 232, 113858.

[6] Z. Sefidgar , A. Ashrafizadeh , A. Arabkoohsar , J. Renew. New Energy 2022, 9, 158.

[7] W. Tian , Z. Mao , H. Ding , Energy Convers. Manage. 2019, 183, 732.

[8] M. Heragy , T. Kono , T. Kiwata , Energy Convers. Manage. 2022, 255, 115326.

[9] F. Fazelpour , N. Soltani , M. Shariatzadeh , M. A. Rosen , in 2015 IEEE 15th International Conference on Environment and Electrical Engineering (EEEIC) , IEEE, Piscataway, NJ 2015, pp. 1587–1592.

[10] M. Baneshi , F. Hadianfard , Energy Convers. Manage. 2016, 127, 233.

[11] M. Qolipour , A. Mostafaeipour , S. Shamshirband , O. Alavi , H. Goudarzi , D. Petkovic , Energy Convers. Manage. 2016, 118, 295.

[12] M. Mohammadi , R. Ghasempour , F. Razi Astaraei , E. Ahmadi , A. Aligholian , A. Toopshekan , Int. J. Electr. Power Energy Syst. 2018, 96, 261.

[13] M. Akbari Vakilabadi , A. Afzalabadi , A. Khoeini Poorfar , A. Rahbari , M. Bidi , M. H. Ahmadi , T. Ming , Int. J. Low‐Carbon Technol. 2019, 14, 10.

[14] S. Ashrafi Goudarzi , F. Fazelpour , G. B. Gharehpetian , M. A. Rosen , Environ. Prog. Sustainable Energy 2019, 38, 13209.

[15] M. A. Abbaszadeh , M. J. Ghourichaei , F. Mohammadkhani , Environ Prog Sustain Energy 2020, 39, e13396.

[16] S. Farahi , F. Fazelpour , Environ. Prog. Sustainable Energy 2019, 38, 614.

[17] O. Rodriguez‐Hernandez , M. Martinez , C. Lopez‐Villalobos , H. Garcia , R. Campos‐Amezcua , Energies 2019, 12, 890.

[18] M. Borunda , J. De La Cruz , R. Garduno‐Ramirez , A. Nicholson , PLoS One 2020, 15, e0230122. 32163479 10.1371/journal.pone.0230122 PMC7067485

[19] I. H. Ibrik , Int. J. Energy Econ. Policy 2019, 9, 26.

[20] O. Ozgener , Energy 2010, 35, 262.

[21] F. Mahmuddin , N. Puspitasari , M. U. Pawara , Earth Environ. Sci. 2021, 921, 12030.

[22] M. Abdelrahim , G. Alkawsi , A. A. Alkahtani , A. M. W. Alhasan , M. Khudari , M. R. Abdul Kadir , J. Ekanayake , S. K. Tiong , Energies 2022, 15, 5412.

[23] A. Rahimi , M. Thaghafi , Environ. Sci. Technol. 2006, 8, 79.

[24] R. Shahbazi , S. Koravand , S. R. Hasan Begi , Reg. Sci. Coop. Dev. Congr. Food Ind. Agric. Sci. 2017.

[25] M. J. Mehrani , F. Arab Markadeh , F. Asadpour , M. A. Ashraf , in First National Environment Conference , 2014.

[26] S. Kuravand , J. Soc. Mech. Eng. 2018, 27, 32.

[27] Electricity tariffs and their general conditions (applicable from 04.21.2021), Ministry of Energy, 2021.

[28] Guaranteed electricity purchase tariffs from renewables, *Renewable Energy Organization*, http://www.satba.gov.ir/fa/guidance/guidance (accessed: October 2021).

[29] *Enactment to guarantee the purchase of electricity produced by renewable and clean power plants. No. 1400/15224/20/100 dated 5.15.2021 (in persian)*.

[30] *Enactment to guarantee the purchase of electricity produced by renewable and clean power plants. No. 1398/33560/20/100 dated 10.20.2019 (in persian)*.

[31] *Announcement of the guaranteed purchase price of electricity from renewable and clean power plants. No. 95/14273/30/100 dated 05.08.2016 (in persian)*.

[32] *Announcement of the guaranteed purchase price of electricity from renewable and clean power plants. No. 94/23773/60/100 dated 07.21.2015 (in persian)*.

[33] Germany electricity prices, December 2021 | GlobalPetrolPrices.com, https://www.globalpetrolprices.com/Germany/electricity_prices/ (accessed: August 2022).

[34] “Germany: household electricity prices 2020 |Statista.” https://www.statista.com/statistics/418078/electricity-prices-for-households-in-germany/ (accessed: August 2022).

[35] German onshore wind power – output, business and perspectives | Clean Energy Wire, https://www.cleanenergywire.org/factsheets/german-onshore-wind-power-output-business-and-perspectives (accessed: August 2022).

[36] H. E. Beck , N. E. Zimmermann , T. R. Mcvicar , N. Vergopolan , A. Berg , E. F. Wood , Sci. Data 2018, 5, 180214. 30375988 10.1038/sdata.2018.214 PMC6207062

[37] LWS micro turbine til tag eller hjørne montage 50 | Campen Auktioner A/S, https://campenauktioner.hibid.com/lot/36479802/lws-micro-turbine-til-tag-eller-hj-rne-montage-50/?q=&ref=catalog (accessed: August 2021).

[38] Amazon.com: Wind Charge Controller 12 V/24 V 300 W/600 W, https://www.amazon.com/Controller-Waterproof-Turbine-Generator-Regulator/dp/B07RFF6PF7/ref=sr_1_12?crid=LFA28M98AVZY&dchild=1&keywords=wind+turbine+charge+controller&qid=1626543735&sprefix=Wind+turbine+controller+charge%2Caps%2C480&sr=8-1.2 (accessed: August 2021).

[39] R. Maouedj , A. Mammeri , M. D. Draou , B. Benyoucef , Energy Procedia 2015, 74, 1192.

[40] A. Behzadi , A. Arabkoohsar , Y. Yang , Appl. Therm. Eng. 2020, 181, 115926.

[41] W. Shen , X.i Chen , J. Qiu , J. A. Hayward , S. Sayeef , P. Osman , K.e Meng , Z. Y. Dong , Renew. Sustainable Energy Rev. 2020, 133, 110301.

[42] Guide to using the resource protection user panel with the development of renewable energy and the efficiency of electricity. Bureau of Social, Economic and Environmental Studies of Satba, 2019, p. 4.

[43] S. Katoch , S. S. Chauhan , V. Kumar , Multimed. Tools Appl. 2021, 80, 8091. 33162782 10.1007/s11042-020-10139-6 PMC7599983

[44] C. A. C. Coello , G. B. Lamont , D. A. Van Veldhuizen , Evolutionary Algorithms for Solving MultiObjective Problems, Springer US, Boston, MA 2007.

[45] C. J. Fourie , W. J. Perold , IEEE Trans. Appl. Supercond. 2003, 13, 511.

[46] K. Deb , A. Pratap , S. Agarwal , T. Meyarivan , IEEE Trans. Evol. Comput. 2002, 6, 182.

[47] A. Gambier , in 2008 American Control Conference, 2008, IEEE, Piscataway, NJ, p. 4727, 10.1109/ACC.2008.4587241.

[48] K. Ahmed , P. Pylsy , J. Kurnitski , Sol. Energy 2016, 137, 516.

[49] M. Sepehr , R. Eghtedaei , A. Toolabimoghadam , Y. Noorollahi , M. Mohammadi , Sustainable Cities Soc. 2018, 41, 481.

[50] A. Marszal‐Pomianowska , P. Heiselberg , O. Kalyanova Larsen , Energy 2016, 103, 487.

[51] Low wind flow systems, 2015, www.think-renewable.at (accessed: August 2021).

